# Editorial: Veterinary Bacterial Zoonoses, volume II

**DOI:** 10.3389/fvets.2023.1245623

**Published:** 2023-07-13

**Authors:** Peng Li, Jiabo Ding, Ting Xin, Shengqing Yu, Menachem Banai

**Affiliations:** ^1^Institute of Animal Sciences, Chinese Academy of Agricultural Sciences, Beijing, China; ^2^Shanghai Veterinary Research Institute, Chinese Academy of Agricultural Sciences, Shanghai, China; ^3^Department of Bacteriology, Kimron Veterinary Institute, Bet Dagan, Israel

**Keywords:** bacterial zoonoses, brucellosis, coxiellosis, bovine tuberculosis, vaccine

Bacterial zoonoses caused by *Brucella, Coxiella*, and *Mycobacterium* are widespread contagious bacterial diseases that are naturally transmissible between animals and people. The epidemic of these zoonotic diseases is becoming more and more serious and complex, resulting in many animals and people around the world being affected every year, posing a risk to the close relationship between animals and people. As a result, it is important to be aware of the common approach to preventing and controlling these bacterial zoonoses. One Health is a collaborative, multisectoral, and transdisciplinary approach taken by the community, and it is gaining recognition globally as an effective way to fight bacterial zoonoses at the human–animal–environment interface.

This Research Topic aims to collect the latest discoveries on brucellosis, coxiellosis, and tuberculosis, including the prevalence situation in the livestock population, available prevention and control measures, detailed characteristics of the causative pathogens, and host immune responses induced by the vaccines of these bacterial zoonoses, which will facilitate the prevention and control of these bacterial zoonoses via the One Health approach. A total of 15 manuscripts were received, 5 of which were published in our Research Topic. Two original research papers and one review paper focused on the prevalence of cattle brucellosis, the genome characteristics of *Brucella* vaccine strains, and the advances of different types of brucellosis vaccines as the available prevention and control measures. One original research paper focused on host immune responses induced by the *Coxiella burnetii* phase I vaccine, and another original research paper focused on the immune responses in granulomas of cattle naturally infected with *Mycobacterium bovis*.

Brucellosis, also known as “Malta Fever”, caused by a Gram-negative bacterium, *Brucella* spp., is a common zoonosis affecting half a million people annually and all domestic animals (1). The prevalence of brucellosis in cattle at different geographical areas has been extensively investigated over the past decades, which is of great significance in terms of epidemiological dimensions (2). In our Research Topic, Ntivuguruzwa et al. studied the epidemiology of brucellosis caused by *B. abortus* and *B. melitensis* in cattle in Rwanda, indicating that brucellosis in cattle is prevalent and threatens the health of handlers of live cattle and carcasses and consumers of unpasteurized milk and milk products. To date, the most effective control strategy for preventing the spread of brucellosis in high-prevalence regions is vaccination (3). Several live-attenuated vaccines (Rev.1, S19, and RB51) have been licensed for preventing animal brucellosis, but no safe and effective vaccine has been licensed for preventing human brucellosis so far (4). In our Research Topic, Heidary et al. summarized the advances of the different types of brucellosis vaccines (live-attenuated vaccines, vector-based vaccines, subunit vaccines, DNA-based vaccines, nanoparticles-based vaccines, and other vaccines), thus providing a more comprehensive understanding of them. *Brucella* spp. virulence relies mainly on its ability to invade and replicate within professional and non-professional phagocytes (5, 6). Many virulence factors, such as lipopolysaccharide (LPS), the type IV secretion system (T4SS), a two-component regulatory system (BvrS/BvrR), and cyclic β-1,2-glucan (CβG), have been identified (7). In our Research Topic, Prasolova et al. characterized phylogenetic relationships in vaccine strains from a Russian Research Topic, identifying some candidate mutations in virulence genes that were responsible for the attenuated virulence of vaccine strains. These studies provide a theoretical basis for developing new brucellosis vaccines and elucidate the pathogenic mechanism of *Brucella*.

Coxiellosis (“Q fever” in humans) caused by the agent *C. burnetii* is a worldwide zoonosis (8). This disease is most commonly transmitted by breathing infectious aerosols or dust contaminated with birth fluids of domestic ruminants (9). In several European countries, a formalin-inactivated whole-cell vaccine (*C. burnetii* phase I) is licensed for cattle and goats (10). In our Research Topic, Bauer et al. evaluated host immune responses induced by the *Coxiella burnetii* phase I vaccine and the influence of the *C. burnetii* phase I vaccine on perinatal complications in ewes and lambs, thus providing more information about the *C. burnetii* phase I vaccine.

Bovine tuberculosis caused by *M. bovis* is a chronic granulomatous caseous-necrotizing inflammatory process affecting different mammals, including humans (11). However, the immune response presented in the granulomas of animals naturally infected with *M. bovis* is unknown. In our Research Topic, Carrisoza-Urbina et al. characterized the immune response in granulomas from adult cattle and calves, demonstrating that granulomas from calves exhibited a greater proinflammatory response and a lack of connective tissue capsule compared to the granulomas from adult cattle, implying that the immune responses in granulomas of cattle may be age dependent. This study helps to understand the immune response of granulomas induced by *M. bovis* at the cellular and molecular levels.

Taken together, the prevalence of cattle brucellosis, the genome characteristics of *Brucella* vaccine strains, the advances of different types of brucellosis vaccines, the host immune responses induced by the *Coxiella burnetii* phase I vaccine, and the immune response of granulomas induced by *M. bovis* were addressed in our Research Topic, which is of great significance for the prevention and control of these bacterial zoonoses.

## Author contributions

PL and JD drafted the manuscript. SY, TX, and MB revised the manuscript. All authors read and approved the final manuscript.
